# Body composition and bone mass among 5-year-old survivors of necrotizing enterocolitis

**DOI:** 10.1038/s41390-022-02236-z

**Published:** 2022-08-16

**Authors:** Amanda Magnusson, Diana Swolin-Eide, Anders Elfvin

**Affiliations:** 1 Department of Pediatrics, Institution of Clinical Sciences, Sahlgrenska Academy, University of Gothenburg, Gothenburg, Sweden; 2 Region Västra Götaland, Department of Pediatrics, The Queen Silvia Children’s Hospital, Sahlgrenska University Hospital, Gothenburg, Sweden

## Abstract

**Background:**

Necrotizing enterocolitis (NEC) affects the intestine of preterm infants. Preterm infants risk inadequate bone mineralization. This risk may increase if the intestinal uptake of minerals is affected after NEC.

**Methods:**

This is a study of growth, bone mineral density (BMD), bone mineral content (BMC), and body composition at 5 years of age among Swedish children born before gestational week 37 + 0 with a history of NEC, minimum stage IIA, compared to matched controls. Fifty children, 25 NEC cases and 25 controls, were examined with dual energy X-ray absorptiometry (DXA) and DXA with laser.

**Results:**

The NEC cases had lower weight, −1.3 SDS vs −0.7 SDS, a lower fat mass and fat percent, 23.4 vs 29.1%, compared to the controls. NEC cases had lower BMC total body head excluded, 355.6 g vs 416.7 g. BMD *Z*-scores were lower among NEC cases in total body head excluded, −0.7 vs −0.1, and in lumbar spine.

**Conclusions:**

Preterm NEC survivors at 5 years of age had reduced growth, an altered body composition, and indications of a lower bone mass compared to matched controls. The study suggests that preterm infants diagnosed with NEC need special attention during childhood regarding growth and bone health.

**Impact:**

- A follow-up longitudinal study of growth, bone health, and body composition at 5 years of age among children born preterm with a history of NEC compared to matched controls.
- The NEC cases had lower weight than controls.
- NEC cases had an altered body composition with lower fat mass compared to controls.
- The DXA results showed that the NEC cases had lower bone mineral content and a tendency to lower bone mineral density.
- The study suggests that preterm infants diagnosed with NEC need special attention at follow-up regarding growth and bone health compared to preterm infants without NEC.

## Introduction

Necrotizing enterocolitis (NEC) is one of the most common life-threatening gastrointestinal diseases in neonates, primarily affecting infants born preterm.^1^ A majority of the NEC cases are treated medically, but 20–40% require surgery.^2,3^ Improvements in neonatal care have increased the overall survival of preterm infants.^4^ However, mortality in NEC has not decreased significantly over the years and remains high.^1,3,5,6^ Among the survivors, there is a major risk for long-term morbidity with both neurodevelopmental and gastrointestinal impairments.^4,6,7^ Infants with NEC have an increased risk of developing intestinal failure. In most cases, the intestinal failure will resolve.^8,9^ In some cases, typically after extensive intestinal surgery, short bowel syndrome (SBS) will develop. This can lead to inadequate absorption of nutrients and affected long-term growth.^3,4,9^ Some studies have suggested that preterm infants with medically managed NEC have long-term outcomes equal to preterm infants without NEC.^10,11^ Others however have shown that children and adolescents with a history of NEC have a slower growth rate compared to controls without NEC.^12^

During the third trimester of pregnancy, the mineralization of bone mainly occurs, where 80% of a term infant’s body calcium is accrued and, accordingly, infants born preterm miss a major part of the fetal bone development. Hence, preterm infants are at risk of inadequate bone mineralization and development of osteopenia, and potentially also osteoporosis, later in life.^13^ We have previously shown that children with a history of NEC have an increased risk of being diagnosed with Rickets early in life compared to children without NEC. We could not show any increased risk of fractures during adolescence following neonatal NEC.^14^ There is a lack of studies of bone mass during childhood among children born preterm with a history of NEC. The aim of this study was to test the hypothesis that children born preterm with a history of NEC, both medically and surgically treated, have reduced growth, reduced bone mass, and an altered body composition compared to matched controls at 5 years of age.

## Methods

This was a study of growth, bone health, and body composition among preterm infants with a history of NEC who were 5 years of age at inclusion in the study. Children born before 37 gestational weeks who developed NEC during their neonatal period and were treated for their NEC at the Queen Silvia Children’s Hospital, Gothenburg, Sweden were included. All children who developed a minimum of stage IIa NEC according to the Bell’s staging criteria modified by Walsh and Kliegman were eligible for inclusion.^15^ Both medically treated and surgically treated NEC was included. Children were not included if deceased before 5 years of age, had another major intestinal disease, had emigrated at 5 years of age, declined to participate in the study, did not respond to the request for participation, or did not show up at the study visit. Twenty-five children with a history of NEC (hereinafter referred to as NEC cases), born between April 2008 and November 2011, were included.

**Fig. 1:**
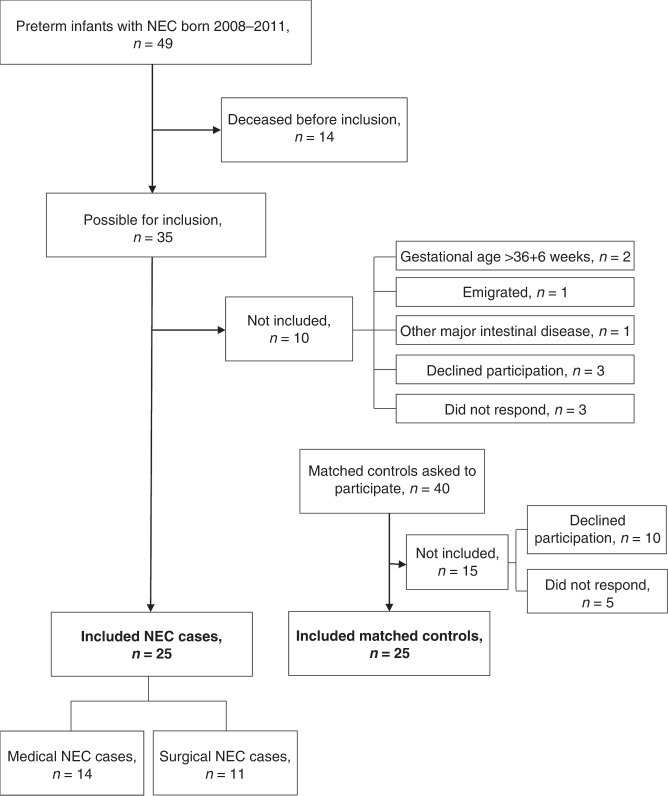
Flow chart over included NEC cases and controls. Each box represents a presentation of included or not included cases or controls, and the reason for not being included.

During the study period, a total of 1619 infants born before gestational week 37 + 0 were admitted to the neonatal intensive care unit (NICU) in Gothenburg, Sweden. Of these 1619 infants, 274 were born before gestational week 28 + 0. As shown in Fig. 1, 49 NEC cases were treated in the NICU during 2008–2011. In total, 24 children with a history of NEC were not included for the following reasons: 14 deceased, 2 with gestational age >36 + 6 weeks, 1 with Hirschsprung’s disease, 1 had emigrated, 3 declined participation, and 3 did not respond or did not show up at the planned visit (Fig. 1). For every NEC case, a control child was included (hereinafter referred to as controls), matched for gestational age, sex, and age at the time for measurement with dual energy X-ray absorptiometry (DXA) but who had not developed NEC during the neonatal period. Of the controls invited for participation, 10 declined participation and 5 did not respond or did not show up at the planned visit. New matched controls were invited until the number of matched controls was 25 (Fig. 1). All controls were born between April 2009 and 2 April 2012, except one who was born in December 2014. In total, 50 children were included in the study.

### Definitions

Patent ductus arteriosus (PDA) was defined as an open ductus arteriosus treated with medicine and/or surgery. Indomethacin was used for medical treatment. Children treated surgically for PDA underwent surgery either as first choice or after indomethacin treatment. In some of the cases with surgical NEC, PDA surgery was done at the same occasion as the NEC surgery. Bronchopulmonary dysplasia (BPD) was defined as requiring supplemental oxygen at 36 weeks of corrected gestational age, and severe BPD as requiring ≥30% oxygen and/or continuous positive airway pressure/ventilator treatment at 36 weeks of corrected gestational age. There are four stages of intraventricular hemorrhage (IVH), and severe IVH was here defined as a minimum of stage 3. Retinopathy of prematurity (ROP) is classified in five stages. Severe ROP was defined as a minimum of stage 3. Abdominal surgery was defined as surgery through the abdominal wall, for example, laparotomy or laparoscopy.

### Data collection

Birth data, infant characteristics from the neonatal period, and data from surgical events were collected from medical records. Diagnoses of concern, for example, IVH, BPD, and ROP, were found in the medical records and the Swedish Neonatal Quality Register. Questionnaires were sent to caregivers, including questions regarding the child’s potential medical conditions, medication, surgeries, lifestyle, nutrition, vitamin D intake, fractures, and family history of bone disease. Data on enteral and parenteral nutrition during the neonatal period were collected from the medical records. Growth data from the children’s first 5 years of life were collected from the medical records and from growth charts from the children’s child care centers.

### Collection of bone mass and body composition

Body height and weight were measured for all children with the same calibrated scale and by the same trained nurse at Queen Silvia Children’s Hospital. Body mass index (BMI) was calculated from these data. The measurements were compared to reference values.^16^ At the same appointment areal bone mineral density (BMD) (g/cm^2^), bone mineral content (BMC) (g), and body composition were measured with DXA (Lunar Prodigy, GE Lunar Corp., Madison, WI) for total body head excluded (TBHE), hip, lumbar spine (LS) (L_1_–L_4)_, trunk, left arm, and left leg. Areal BMD is the bone mineral content, measured by the two-dimensional method DXA, divided by the bone area in square centimeters. *Z*-scores for BMD from the Lunar international reference database were used as previously described.^17^ From 2016 onwards, the DXA measurements were made on Lunar iDXA (GE Lunar Corp., Madison, WI). To compare the results from Lunar Prodigy DXA with Lunar iDXA, a reliability study has previously been described.^17^ The reliability was evaluated as acceptable based on high intraclass correlation coefficient and low coefficient of variation. Calcaneal BMD and BMC were assessed in the left foot using the DXL Calscan technique (Scanflex/Demetech AB, Täby, Sweden), which is a combination of DXA and a laser measurement. Mainly, the trabecular bone of the calcaneus is more reactive to metabolic changes.^18^ The DXL Calscan technique has been modified for pediatric use, which is described elsewhere.^19^ A function enabling manual measurement of the calcaneal height is available in this pediatric version of the DXL Calscan. This calcaneal height measurement together with the calcaneal BMD value enables calculation of the volumetric bone mineral apparent density (BMAD) (g/cm^3^). Areal BMD measured by DXA is referred to as BMD (g/cm^2^), and volumetric BMAD is referred to as BMAD (g/cm^3^) in the present study. Comparing BMD values can be misleading when comparing values from different sizes of bones; therefore, BMAD is valuable when evaluating BMD of persons of different height and growing individuals. The BMC, BMD, and BMAD values from the DXL were compared to the DXL reference group of healthy Swedish children at 4–5 years of age where the values are presented in percentiles.^19^

In children, the head represents a large portion of the total bone mass and thus should be excluded; otherwise, it could disguise deficits in bone mass at other sites in the body.^20,21^ In this study, the scans of TBHE were evaluated.

*Z*-scores were in this study available for BMD of TBHE, LS, and the hips.

The children were not sedated for the DXA or DXL measurements. All the measurements were made by the same trained nurse and analyzed by the same expert in pediatric densitometry. The nurse did not know the group belonging of the individual child.

### Statistics

A power calculation was made in collaboration with Statistiska konsultgruppen in Gothenburg. The desired power was set to 0.8. The groups selected for the power calculation were the children with a history of medical or surgical NEC (all NEC cases) and their matched controls. The primary outcome for the power calculation was BMC (TBHE). Every NEC case was matched with a control child without NEC, matched for gestational age, sex, and age at the time for the DXA measurement. Therefore, it can be assumed that there was a relatively good similarity between the groups. We assumed the median and mean TBHE BMC to be between 300 and 500 g with an effect size of at least 25 g difference between the groups. According to the power calculation, 25 children in each group, 50 children in total, were needed to detect significant differences between the groups.

Data were presented as number (*n*) and percentage (%) for categorical data and as median (min; max) and mean (95% confidence interval (CI)) for continuous data. Variable differences were analyzed with Chi-square test and Fisher’s exact test for categorical data. For quantitative data, variable differences were analyzed using Mann–Whitney *U*-test. A multivariable linear regression was performed to correct for height as a confounding factor on BMC. The statistical significance level was set as *p* < 0.05. All statistical analyses were calculated with SPSS Statistics, version 26 (IBM Corp., Armonk, NY).

## Results

Neonatal characteristics of the 25 NEC cases and the 25 matched controls are presented in Table 1. There were no differences between NEC cases and controls regarding gestational age, birth weight, birth length, or head circumference. Furthermore, there were no differences between the groups regarding BPD, PDA, severe IVH, or severe ROP (Table 1).

**Table 1:** Neonatal characteristics of NEC cases and controls.

Infant characteristics	NEC *n* = 25	Controls *n* = 25	*p* value
Gestational age, weeks	27 (24; 35)	28 (24; 35)	0.72
Gestational age, days	190 (168; 254)	196 (168; 246)	0.92
Female sex, *n*	14 (56%)	14 (56%)	1
Multiple gestation, *n*	12 (48%)	9 (36%)	0.57
Cesarean delivery, *n*	17 (68%)	11 (44%)	0.15
Birth weight, g	945 (470; 2320)	1095 (465; 2465)	0.25
Birth length, cm	35 (28; 48)	37 (29; 47)	0.33
Head circumference, cm^a^	25.0 (20.4; 32.0)	25.8 (20.6; 32.5)	0.3
PDA, surgical treatment, *n*	7 (28%)	3 (12%)	0.29
PDA, medical treatment, *n*	11 (44%)	10 (40%)	1
Any BPD^a^, *n*	8 (33%)	12 (48%)	0.39
Severe BPD^a^, *n*	2 (8%)	1 (4%)	0.61
IVH grade 3–4^b^, *n*	2 (8%)	2 (8%)	1
ROP stage 3–5^b^, *n*	7 (28%)	5 (20%)	0.74
Abdominal surgery, *n*	17^c^ (68%)	1 (4%)	<0.001
Total number of days on PN median (min; max)	30 (9; 216)	7 (0; 21)	<0.01

Median (min; max) for continuous variables, number (%) for categorical variables. Mann–Whitney *U*-test was used for continuous data. Fisher’s exact test was used for categorical data.

*PDA* patent ductus arteriosus, *BPD* bronchopulmonary dysplasia, *IVH* intraventricular hemorrhage, *ROP* retinopathy of prematurity, *PN* parenteral nutrition.

^a^Value missing from one NEC case.

^b^Values missing for one NEC case and one control.

^c^11 surgical NEC, 5 medical NEC with surgery later due to suspect post-NEC ileus and 1 medical NEC with resection of the gall bladder.

### Nutritional data during first admission

All infants received at least minimal enteral feeding with mother’s own milk or pasteurized donor milk from the first day of life. Enteral feeding was increased as tolerated with mother’s own milk if available, and donor milk if there was not enough mother’s own milk. None of the infants received any formula feeding before gestational age of 35 + 0 weeks. Bovine-based fortifier was added to the milk when enteral feeding was between 70 and 100 ml/kg/day. A few of the NEC patients with an early onset of disease developed NEC before initiation of fortifier. There was no difference between the groups regarding the initiation of feeding. Parenteral nutrition was initiated during the first day of life in 22/25 controls, and in 24/25 of the NEC cases. Three of the control infants with gestational age at birth >31 + 0 weeks had no days on parenteral nutrition. The controls received parenteral nutrition for a median of seven days with a range of 0–21 days (Table 1).

In the NEC group, all infants had at least one episode of parenteral nutrition. The range in total number of days on parenteral nutrition during the first NICU admission among the NEC cases varied from 9 days to 216 days, with a median of 30 days (Table 1). Four of the NEC infants had parenteral nutrition >100 days.

None of the infants in the study received any probiotics during the first admission to the NICU.

### NEC characteristics

The median age at NEC diagnosis was 18 days with a range between 1 day of age to 37 days of age. Mean age at NEC diagnosis was 20 days. Regarding the patient with NEC diagnosis at day 1 of life, there was a discussion with the pediatric surgeon regarding spontaneous intestinal perforation as a possible differential diagnosis, but the NEC diagnosis remained. Eleven (44%) of the 25 NEC cases needed surgical intervention for their NEC (hereinafter referred to as surgical NEC cases), and 14 were treated medically (medical NEC cases). Of the 14 medical NEC cases, five needed abdominal surgery at a later occasion due to suspected post-NEC ileus. In total, 17 (68%) NEC cases had at least one abdominal surgery: 11 surgical NEC cases, 5 medical NEC cases with surgery later due to suspicious post-NEC ileus, and 1 medical NEC case with resection of the gall bladder. One child underwent laparotomy seven times. Of the controls, one underwent abdominal surgery due to a ruptured appendix. Surgical findings are presented in Supplementary Table 1.

### Growth

At the time of DXA measurement, the median age for both NEC cases and controls was 5.1 years old. As shown in Table 2, the NEC cases were shorter and had lower weight compared to controls. Furthermore, when compared to reference values for healthy children, the weight standard deviation (SDS) was lower among NEC cases compared to controls. No difference was found between the groups regarding height SDS when compared to reference values for healthy children, but both groups were below zero, −1.1 SDS for NEC cases and −1.0 SDS for controls.^16^ Regarding the surgical NEC cases, both the weight SDS and the height SDS were lower among surgical NEC cases compared to controls. No difference in BMI was found between the groups (Table 2).

**Table 2:** Age and growth-characteristics.

Variable	All NEC *n* = 25; median (min; max)	All controls *n* = 25; median (min; max)	*p* value	Surgical NEC *n* = 11; median (min; max)	Controls *n* = 11; median (min; max)	*p* value
Age, years	5.1 (4.3; 6.0)	5.1 (4.5; 5.9)	0.648	5.1 (4.4; 6.0)	5.3 (4.5; 5.9)	0.478
Weight, kg	16.6 (13.2; 23.0)	18.3 (12.1; 22.9)	**0.006**	16.6 (13.2; 18.0)	18.3 (16.4; 21.2)	**0.004**
Weight SDS	−1.3 (−3.2; 0.7)	−0.7 (−3.7; 1.3)	**0.014**	−1.3 (−3.2; 0.6)	−0.7 (−1.7; 0.2)	**0.028**
Height, cm	106.5 (98.7; 119.7)	108.6 (96.2; 117.9)	**0.041**	106.8 (102.0;110.3)	110.5 (106.2; 117.9)	**0.002**
Height SDS	−1.1 (−2.6; 0.6)	−1.0 (−2.8; 1.0)	0.105	−1.3 (−2.0; −0.3)	−0.5 (−1.3; 0.6)	**0.013**
BMI, kg/m^2^	14.7 (12.3; 16.2)	15.2 (13.0; 17.6)	0.159	14.7 (12.3; 15.7)	15.0 (14.0; 16.1)	0.217
BMI SDS	−0.8 (−3.2; 0.5)	−0.4 (−3.1; 1.4)	0.123	−0.8 (−3.2; 0.2)	−0.4 (−1.7; 0.5)	0.082
Maternal height (cm)	165 (156; 173)	165 (155; 170)	0.71			
Paternal height (cm)	180 (174; 194)	180 (161; 189)	0.43			
Mid parental height SDS	−0.1 (−1.1; 1.6)	−0.2 (−1.8; 0.9)	0.96			
Target height SDS	−0.2 (−1.1; 1.3)	0.0 (−1.7; 0.7)	0.92			

Values are presented in median (min; max). Data regarding maternal and paternal height were collected from questionnaire.

*p*-values in bold represent a *p* < 0.05.

### Bone and body composition measurements

Body composition calculations were made on values from TBHE, trunk, left leg, and left arm. Calculations of BMC and BMD were made on values from TBHE, LS, hip (mean value for right and left hip), trunk, left leg, and left arm. As shown by the variations in (*n*) in Tables 3 and 4, a number of scans were excluded due to movement artifacts, and therefore unreliable values. Consequently, the number of values accounted for are in some calculations lower than the total number of included children (Tables 3 and 4).

**Table 3:** Body composition measured by DXA.

Variable	All NEC *n* = 25; median (min; max)	All controls *n* = 25; median (min; max)	*p* value
TBHE lean mass (kg)	*n* = 21 9.99 (7.78; 14.52)	*n* = 24 10.30 (8.97; 13.54)	0.57
TBHE fat mass (kg)	*n* = 21 3.11 (1.89; 5.05)	*n* = 24 4.51 (2.71; 5.85)	**0.001**
TBHE fat percent (%)	*n* = 21 23.4 (13.6; 34.0)	*n* = 24 29.1 (19.8; 39.4)	**0.007**
Trunk lean mass (kg)	*n* = 24 5.95 (4.85; 8.00)	*n* = 25 5.98 (4.53; 7.68)	1.0
Trunk fat mass (kg)	*n* = 24 1.25 (0.53; 2.01)	*n* = 25 1.68 (0.87; 2.52)	**0.002**
Trunk fat percent (%)	*n* = 24 17.0 (7.8; 28.3)	*n* = 25 22.6 (11.9; 31.0)	**0.005**
Left leg lean mass (kg)	*n* = 22 1.54 (1.00; 2.39)	*n* = 24 1.60 (1.28; 2.14)	0.442
Left leg fat mass (kg)	*n* = 22 0.67 (0.34; 1.08)	*n* = 24 0.97 (0.66; 1.41	<**0.001**
Left leg fat percent (%)	*n* = 22 32.2 (17.4; 44.8)	*n* = 24 39.0 (28.8; 47.4)	**0.002**
Left arm lean mass (kg)	*n* = 21 0.51 (0.37; 0.77)	*n* = 24 0.54 (0.45; 0.71)	0.275
Left arm fat mass (kg)	*n* = 21 0.25 (0.06; 0.46)	*n* = 24 0.33 (0.19; 0.48)	**0.01**
Left arm fat percent (%)	*n* = 21 31.1 (9.7; 47.5)	*n* = 24 35.3 (25.5; 48.7)	0.064

Values are presented as median (min; max).

*TBHE* total body head excluded.

*p*-values in bold represent a *p* < 0.05.

**Table 4:** Bone mass measured by DXA.

Variable	All NEC *n* = 25; median (min; max)	All controls *n* = 25; median (min; max)	*p* value (*p* value after correction for height at the time for DXA/DXL)
TBHE BMC (g)	*n* = 21 355.6 (230.3; 531.6)	*n* = 24 416.7 (310.0; 500.7)	**0.001** **(0.003)**
LS BMC (g)	*n* = 24 12.2 (9.5; 17.7)	*n* = 25 13.7 (10.0;17.4)	**0.028** (0,151)
Hip total BMC (g)	*n* = 24 5.2 (3.1; 8.7)	*n* = 24 5.8 (3.4; 8.2)	0.112
Trunk BMC (g)	*n* = 24 155.4 (116.3; 216.2)	*n* = 25 174.9 (133.7; 228.1)	**0.003** **(0.004)**
Left leg BMC (g)	*n* = 22 69.2 (43.3; 110.8)	*n* = 24 85.3 (45.9; 105.1)	**0.002** **(0.017)**
Left arm BMC (g)	*n* = 21 26.6 (14.3; 43.9)	*n* = 24 32.8 (24.2; 42.1)	**0.001** **(0.014)**
TBHE BMD (g/cm^2^)	*n* = 21 0.490 (0.401; 0.640)	*n* = 24 0.508 (0.414; 0.582)	0.666
LS BMD (g/cm^2^)	*n* = 24 0.563 (0.438; 0.688)	*n* = 25 0.586 (0.477; 0.778)	0.085
Hip total BMD (g/cm^2^)	*n* = 24 0.574 (0.436; 0.655)	*n* = 25 0.592 (0.484; 0.726)	0.332
Trunk BMD (g/cm^2^)	*n* = 24 0.498 (0.398; 0.612)	*n* = 25 0.509 (0.414;0.569)	0.795
Left leg BMD (g/cm^2^)	*n* = 22 0.562 (0.442; 0.708)	*n* = 24 0.562 (0.477; 0.66)	0.636
Left arm BMD (g/cm^2^)	*n* = 21 0.381 (0.344; 0.551)	*n* = 24 0.399 (0.299; 0.517)	0.918
TBHE BMD *Z*-score	*n* = 20 −0.7 (−2.5; 0.4)	*n* = 23 −0.1 (−1.5; 2.2)	**0.015**
LS BMD *Z*-score	*n* = 22 −0.7 (−2.5; 0.8)	*n* = 23 −0.2 (−1.9; 2.1)	**0.037**
Hip total BMD *Z*-score	*n* = 22 −1.1 (−2.5; 0.1)	*n* = 22 −0.8 (−2.5; 1.2)	0.324

Values presented as median (min; max).

*TBHE* total body head excluded, *LS* lumbar spine, *BMC* bone mineral content, *BMD* bone mineral density.

*p*-values in bold represent a *p* < 0.05.

### Body composition

Data on tissue mass are presented in Table 3. Regarding fat mass, as shown in Table 3, the NEC cases had lower fat mass compared to controls in TBHE (3.11 vs 4.51 kg), trunk (1.25 vs 1.68 kg), left leg (0.67 vs 0.97 kg), and left arm (0.25 vs 0.33 kg) compared to controls. Furthermore, the fat percentage was significantly lower among the NEC cases in TBHE (23.4 vs 29.1%), trunk (17.0 vs 22.6%), and the left leg (32.2 vs 39.0%). No differences were found between the groups regarding the amount of lean mass in TBHE, trunk, left leg, or left arm (Table 3). Results regarding surgical NEC cases were similar to the findings among all NEC cases compared to controls and are presented in Supplementary Table 2.

### Bone measurements—all NEC cases vs matched controls

The NEC cases had lower BMC in TBHE (355.6 vs 416.7 g), LS (12.2 vs 13.7 g), trunk (155.4 vs 174.9 g), left leg (69.2 vs 85.3 g), and left arm (26.6 vs 32.8 g) compared to the controls. This difference remained significant at all sites except the LS, even after correction for height at the time of DXA measurement (Table 4). No difference was found for BMC in the hip. No significant difference was found between the groups for BMD in TBHE, LS, hip, trunk, left leg, or left arm. However, the BMD *Z*-scores were lower in TBHE and LS among the NEC cases compared to the controls (Table 4).

### Bone measurements—surgical NEC cases versus matched controls

Equally, the surgical NEC cases had lower BMC in TBHE, LS, trunk, left leg, and left arm compared to their matched controls, but no differences were found for BMC in the hip. In this small group of 11 cases and 11 controls, the difference in BMC did not remain significant after correction for height at the time of measurement. This extracted group of surgical NEC cases had lower BMD in the LS compared to their matched controls, (0.565 vs 0.618 g/cm^2^, *p* = 0.029). No differences were found between the groups for BMD in TBHE, hip, trunk, left leg, or left arm. The BMD *Z*-scores were lower in TBHE (−0.8 vs 0.2) and LS (−0.7 vs 0.0) among surgical NEC cases compared to controls (Supplementary Table 3).

### DXL

No differences between the groups were found regarding the length of the left foot or the height of the calcaneus. Equally, there were no differences between the groups in BMC, BMD, or BMAD. Neither were there any differences in the BMC, BMD, or BMAD percentiles (Table 5). As shown in Supplementary Table 4, the height of the calcaneus was lower among the surgical NEC cases compared to controls, but no other significant differences were found in the extracted group of surgical NEC cases compared to controls regarding DXL.

**Table 5:** Bone mass in the left foot measured by DXL.

Variable	All NEC *n* = 25; median (min; max)	All controls *n* = 25; median (min; max)	*p* value
Footlength, cm	16.0 (15.1; 19.5)	16.5 (14.9; 18.5)	0.25
BMC, g	0.147 (0.048; 0.246)	0.163 (0.119; 0.229)	0.09
BMC, percentile^a^	30 (0; 100)	50 (5; 95)	0.10
BMD, g/cm^2^	0.199 (0.084; 0.283)	0.220 (0.159; 0.31)	0.06
BMD, percentile^a^	30 (0; 95)	50 (5; 95)	0.06
Calcaneus height, cm^b^	2.51 (2.14; 3.07)	2.63 (2.17; 3.03)	0.08
BMAD, mg/cm^3,b^	77.6 (39.3; 115.7)	85.7 (54.1; 124.3)	0.11
BMAD, percentile^b^	38 (0; 95)	60 (0; 100)	0.10

Values presented as median (min; max).

*BMC* bone mineral content, *BMD* bone mineral density, *BMAD* bone mineral apparent density.

^a^Magnusson et al.^14^

^b^Value missing for one NEC case.

### Questionnaire—all NEC cases vs matched controls

No differences were found regarding special diet or the number who reported feeding difficulties. Special diets included nutritional beverages, additional fat and protein, gluten- and dairy-free diets, and food and vitamin supplements. As shown in Supplementary Table 5, two of the controls and six of the NEC cases received special diet or dietary treatment. Of the controls, one had a gastrostomy due to feeding problems, and the other had extra nutritional support following poor growth. Among the NEC cases, three had diary free diet, one had gluten- and diary-free diet, and received additional vitamin D, C, K and probiotics. One child received a special diet with hydralized protein and high amounts of MCT-fat. One of the NEC cases received high energy food due to poor growth. Vitamin D supplementation until 2 years of age was provided to 90% of all children. None of the controls and two of the NEC cases reported a fracture. One child suffered a fractured femur and the other child a clavicular fracture. Nine children in each group participated in organized sport at least once a week. All questions had missing answers from one or two children (Supplementary Table 5).

One NEC case had a heart defect (Epstein’s anomaly), while no heart defects were reported among the controls. Three of the NEC cases and two of the controls had asthma. One NEC case and none of the controls had reported allergies. Hyperthyroidism was reported in one NEC case. None of the controls, but one of the NEC cases had a mild cerebral palsy. One NEC case and none of the controls had post-hemorrhagic hydrocephalus. According to the questionnaires, none of the children in either group had a cognitive disorder, but one NEC case was under investigation for possible neurodevelopmental disorder.

None of these reported medical conditions were regarded as exclusion criteria in the study.

According to the medical records, three NEC cases, but no child in the control group, have been followed up by physiotherapist. Six children in the NEC group and one child in the control group have been referred for neuropsychiatric evaluation.

## Discussion

The present study showed that children born preterm and with a history of NEC had lower weight at 5 years of age than the matched controls. NEC cases had an altered body composition with lower amount of fat mass and fat percentage than the controls. The study also revealed that the NEC cases had lower BMC, a difference that partially remained significant after correction for height, and a lower BMD *Z*-score than controls.

Postnatal growth failure among preterm infants is common and may be affected by many things such as gestational age, birth weight, poor nutrition, concurrent diseases, and medication. Some of the children remain small during childhood.^22–24^ Stoltz Sjöström et al. showed a high risk for severe postnatal growth failure among preterm infants. With optimized nutrition early growth failure in these infants may be prevented.^23^ Horemuzova et al. showed, among extremely preterm Swedish infants at term age, that a large proportion of the infants had *Z*-scores below −2.0 for both weight and height.^25^ However, after term age they had a significant catch-up, and at 10 years of age had almost reached an average height and weight compared to the normal population.

In the present study, gestational age, birth weight, and age when measured did not differ between NEC cases and controls. Neither were there any significant differences in diseases related to prematurity, such as BPD, IVH, or ROP. Still, the NEC cases had a lower weight at 5 years of age. This indicates that NEC itself can affect long-term growth negatively. Several of the other medical conditions reported in the questionnaire, such as Epstein’s anomaly, asthma and hyperthyroidism may affect growth, but were not considered as criteria for exclusion in this study. None of these children represented outliers regarding growth or bone mass; however, it cannot be excluded that children in the NEC group with additional conditions may be of extra risk of poor growth and poor bone development. There is inconsistency in previous studies as to whether NEC survivors have affected growth. Malek et al. investigated long-term, up to 20 years, growth outcomes in children born preterm with and without NEC, and they showed that, as in our study, children with NEC had lower weight gain during the follow-up period than children without NEC.^12^ Malek et al. suggest further that there are variations in intestinal physiology among NEC survivors and not only the intestinal length after surgery that affect growth. Soraisham et al. found, in contrast to our study, no significant difference in either weight or height at 3 years of age among NEC cases compared to matched controls.^26^ Hong et al. showed that extremely low birth weight NEC cases had higher rates of severe growth failure at discharge than infants without NEC.^27^

In the present study, the lower fat percentage among NEC cases compared to controls is explained by the lower fat mass and not by an increase in lean mass.

In a systematic review, Johnson et al. demonstrated that preterm infants had a different body composition at term age compared to infants born at term, with significantly lower fat mass, greater fat percentage, and lower fat-free mass, where there was more difference in fat-free mass than fat mass.^28^ They suggested that the greater fat percentage at term age was rather explained by a lower lean mass than by a larger volume of fat mass. Stigson et al. showed no differences in bone mass, but significantly less lean mass and increased fat mass at 4–5 years of age among children born preterm compared to controls born at term.^26^ A recent study by Binder et al demonstrated that a higher fat free mass *Z*-score was related to a larger brain size among preterm infants.^29^ They suggested that high fat free mass *Z*-score may be used as an indicator of protein accretion and brain growth.^29^ In our study, the preterm NEC cases had lower fat mass, and no difference in lean mass; however, we did not compare with children born at term but with matched controls born preterm.

The NEC cases had lower BMC than the controls at all investigated sites except for the hip. When correcting for height at the time of DXA measurement, the difference remained significant at all sites except for the LS. Bachrach et al. implied that children with chronic illnesses often have delayed growth, and this in turn can contribute to lower bone mass.^20^ In other studies, it is stated that BMC depends on bone size, and a lower BMC can therefore reflect a smaller skeleton.^[30,30^ The NEC cases in our study were significantly shorter than the matched controls. However, when our data were corrected for height, the difference in BMC remained, suggesting that the lower BMC among NEC cases cannot be explained solely by their shorter height. Some studies suggest that areal BMD is shown to be more accurate than BMC as a measure of bone health in children, whereas others propose BMC as the preferred measure.^31,30^ Heaney et al. suggest that it is not correct to use BMD in growth studies because it does not include the component of bone accumulation that is related to changes in bone size; the preferred measure is BMC.^32^ The areal BMD takes bone size into consideration, but only two-dimensional; hence, the adjustment of the bone size is not complete.^21,30^ DXA measures areal BMD (g/cm^2^) with height and width of the bone, but not volumetric BMD (g/cm^3^). Regarding BMD, the only significantly lower values were found in the lumbar spine in the group of surgical NEC cases. The results in the present study had been even more compelling if there had been differences between the NEC cases and controls regarding both BMC and BMD. However, when comparing the BMD *Z*-scores, the NEC cases had lower values in TBHE and LS compared to controls. TBHE and LS, which consist mostly of trabecular bone, have been suggested as the preferred sites for DXA measurements in young children.^15,16^ Taking this into account, the present study does show convincing differences in bone mass between the children with a history of NEC and their matched controls.

In the DXL measurements, the median and mean values were consistently lower among the NEC cases compared to controls, however, not significantly.

Two of the NEC cases had reported fractures compared to none of the controls. Factors that can affect a child’s bone mass and fracture risk are, among others, vitamin D insufficiency and physical inactivity.^20^ Almost all children in both groups received vitamin D supplementation until two years of age, as recommended in Sweden, and it was just as many in both groups that participated in organized sports. We have previously presented results not showing any significantly increased risk of fractures during childhood and adolescence in children born preterm and with a history of NEC.^14^ In the present study it was not possible to draw conclusions whether the two cases of fractures were related to poor bone health or not.

### Strengths and weaknesses

A strength of the present study was that each NEC case had a control matched for gestational age at birth, sex, and age at DXA measurements. All DXA and DXL measurements were performed by the same trained nurse. Another strength was the fact that the NEC cases and controls did not have any differences regarding PDA, BPD, ROP, or IVH. The relatively small number of cases and controls in this study is a weakness.

A weakness with a follow-up study is that a number of years have passed since the first NICU admission. All infants included in the present study were born between 2008 and 2011. Many things have happened in the field of nutrition of the preterm infant since then. None of the infants received any probiotics. Awareness of the importance of the composition of early parenteral as well as enteral nutrition has improved over the past 10 years.

## Conclusion

The present study showed that 5-year-old children born preterm and diagnosed with NEC during the neonatal period had lower weight than their matched controls. The DXA measurements showed that the NEC cases had an altered body composition, with lower amount of fat mass and fat percentage than the controls. It also revealed that the NEC cases had lower BMC, a difference that partially remained significant after correction for height, and a tendency to lower BMD than controls.

The study suggests that preterm infants diagnosed with NEC need special attention at follow-up during childhood regarding growth and bone health compared to preterm infants without NEC.

## Data Availability

The datasets generated during and/or analyzed during the current study are available from the corresponding author on reasonable request.

## Supplementary Table

Supplementary Table
