# Supplementary material for: Body composition and bone mass among 5-year-old survivors of necrotizing enterocolitis
Source: Pediatr Res. 2022 Aug 16;93(4):924–31. doi: 10.1038/s41390-022-02236-z (PMC10033411; doi:10.1038/s41390-022-02236-z)
Supplement: Supplementary file 1 — Supplementary Table [file 41390_2022_2236_MOESM1_ESM.pdf]

Supplementary tables for

Body composition and bone mass among five-year-old survivors of necrotizing enterocolitis

Running title: Altered body composition and bone mass following NEC

Amanda Magnusson[1,2], Diana Swolin-Eide[1,2], Anders Elfvin*[1,2]

[1]Department of Pediatrics, Institution of Clinical Sciences, Sahlgrenska Academy, University of Gothenburg, Gothenburg, Sweden

[2]Region Västra Götaland, Department of Pediatrics, The Queen Silvia Children's Hospital, Sahlgrenska University Hospital, Sweden

Supplementary Table 1. Surgical characteristics

Variable	Surgical NEC<br>n = 11	Medical NEC with abdominal surgery later due to suspect post-NEC ileus<br>n = 5
NEC in colon, n	4	2
Intestinal perforation, n	6	2
Ileocecal valve removal, n	5	2
Stoma, n	11	4

n = Number for categorical data.

Supplementary Table 2. Body composition measured by DXA.
Surgical NEC cases vs controls

Variable	Surgical NEC<br>n = 11<br>median (min; max)	Controls<br>n = 11<br>median (min; max)	p-value
TBHE lean mass (kg)	n=9<br>10.60 (7.78; 11.93)	n=11<br>10.48 (9.20; 12.52)	0.882
TBHE fat mass (kg)	n=9<br>3.03 (1.93; 4.13)	n=11<br>4.73 (2.73; 5.85)	**0.003**
TBHE fat percent (%)	n=9<br>22.1 (14.0; 31.9)	n=11<br>30.1 (19.8; 36.8)	**0.038**
Trunk lean mass (kg)	n=10<br>5.99 (4.85; 7.04)	n=11<br>5.98 (5.42; 7.23)	0.756
Trunk fat mass (kg)	n=10<br>1.19 (0.67; 1.87)	n=11<br>1.97 (0.87; 2.52)	**0.006**
Trunk fat percent (%)	n=10<br>16.9 (9.3; 24.2)	n=11<br>23.2 (11.9; 29.7)	**0.016**
Left leg lean mass (kg)	n=9<br>1.55 (1.00; 1.97)	n=11<br>1.60 (1.31; 1.97)	0.552
Left leg fat mass (kg)	n=9<br>0.65 (0.48; 0.88)	n=11<br>1.00 (0.70; 1.34)	**0.001**
Left leg fat percent (%)	n=9<br>33.0 (21.5; 44.8)	n=11<br>39.6 (28.8; 47.1)	**0.031**
Left arm lean mass (kg)	n=9<br>0.53 (0.38; 0.77)	n=11<br>0.54 (0.45; 0.64)	0.824
Left arm fat mass (kg)	n=9<br>0.23 (0.08; 0.34)	n=11<br>0.32 (0.20;0.43)	**0.02**
Left arm fat percent (%)	n=9<br>30.5 (10.7; 40.7)	n=11<br>36.3 (26.8; 46.1)	0.095

Values presented as median (min; max). TBHE = Total body head excluded

Supplementary Table 3. Bone mass measured by DXA.
Surgical NEC cases vs controls

Variable	Surgical NEC<br>n = 11<br>median (min; max)	Controls<br>n = 11<br>median (min; max)	p-value<br>(p-value after correction for height at the time for DXA/DXL)
TBHE BMC (g)	n=9<br>347.9 (259.6; 384.6)	n=11<br>426.0 (310.0; 500.7)	**0.002**<br>(0.065)
LS BMC (g)	n=10<br>12.4 (9.5; 15.1)	n=11<br>14.5 (10.8; 16.7)	**0.013**<br>(0.477)
Hip total BMC (g)	n=10<br>5.5 (3.0; 6.4)	n=11<br>6.1 (3.9; 7.5)	0.072
Trunk BMC (g)	n=10<br>155.4 (116.3; 176.8)	n=11<br>181.4 (133.7; 228.1)	**0.005**<br>(0.328)
Left leg BMC (g)	n=9<br>71.2 (45.1; 75.7)	n=11<br>87.7 (64.5; 105.1)	**0.001**<br>**(0.028)**
Left arm BMC (g)	n=9<br>25.9 (22.6; 31.8)	n=11<br>32.6 (24.2; 42.1)	**0.006**<br>(0.198)
TBHE BMD (g/cm^2)	n=9<br>0.464 (0.401; 0.593)	n=11<br>0.513 (0.414; 0.544)	0.152
LS BMD (g/cm^2)	n=10<br>0.565 (0.438; 0.679)	n=11<br>0.618 (0.477; 0.669)	**0.029**
Hip total BMD (g/cm^2)	n=10<br>0.574 (0.436; 0.649)	n=11<br>0.599 (0.491; 0.664)	0.114
Trunk BMD (g/cm^2)	n=10<br>0.473 (0.398; 0.592)	n=11<br>0.514 (0.414; 0.545)	0.114
Left leg BMD (g/cm^2)	n=9<br>0.506 (0.456; 0.62)	n=11<br>0.572 (0.477; 0.630)	0.152
Left arm BMD (g/cm^2)	n=9<br>0.357 (0.344; 0.551)	n=11<br>0.394 (0.299; 0.413)	0.175
TBHE BMD Z-score	n=9<br>-0.8 (-2.5; 0)	n=10<br>0.2 (-1.5; 0.9)	**0.022**
LS BMD Z-score	n=10<br>-0.7 (-2.5; 0.8)	n=10<br>0 (-1.7; 0.7)	**0.023**
Hip total BMD z-score	n=10<br>-1.1 (-2.3; 0)	n=10<br>-0.6 (-2.0; 0.2)	0.165

Values presented as median (min; max). TBHE = Total body head excluded, LS = lumbar spine, BMC = bone mineral content, BMD, bone mineral density

Supplementary Table 4. Bone mass in the left foot measured by DXL.
Surgical NEC cases vs controls

Variable	Surgical NEC n = 11 median (min; max)	Controls n = 11 median (min; max)	p-value
Footlength, cm	16.1 (15.5; 17.4)	16.6 (15.8; 17.3)	0.17
BMC, g	0.147 (0.048; 0.198)	0.179 (0.133; 0.198)	0.12
BMC, percentile [a]	30 (0; 85)	75 (15; 85)	0.13
BMD, g/cm^2	0.199 (0.084; 0.269)	0.240 (0.18; 0.268)	0.17
BMD, percentile [a]	30 (0; 85)	70 (15; 85)	0.15
Calcaneus height, cm [b]	2.51 (2.14; 2.95)	2.63 (2.45; 3.03)	**0.04**
BMAD, mg/cm^3 [b]	80.9 (39.3; 115.7)	91.3 (68.5; 96.6)	0.37
BMAD, percentile [b]	45 (0; 95)	70 (15; 85)	0.37

Values presented as median (min; max) BMC = bone mineral content, BMD = bone mineral density, BMAD = bone mineral apparent density
[a] reference [14]

Supplementary Table 5. Results from the questionnaire.

Question	All NEC-cases n = 25	Controls n = 25	p-value
Concerns about the child's growth at the child health care center, n/total n (%)	4/24 (17)	3/24 (13)	1
Special diet/dietary treatment, n/total n (%)	6/24 (25)	2/25 (8)	0.14
Difficult to feed /feeding-difficulties, n/total n (%)	11/24 (46)	6/25 (24)	0.14
Vitamin D-supplement until 2 years old, n/total n (%)	22/24 (92)	22/25 (88)	1
History of at least one fracture, n/total n (%)	2/24 (8)	0/25 (0)	0.24
Bone disease in family, n (%)	2/23 (8)	0/25 (0)	0.22
Participation in organized exercise or sport, n (%)	9/23 (39)	9/24 (38)	0.77

Number (%) for categorical data. Fisher's exact test was used for categorical data. Answers missing from 1-2 children among the NEC-cases.
